# Toxicological Evaluation of a Rotenone Derivative in Rodents for Clinical Myocardial Perfusion Imaging

**DOI:** 10.1007/s12012-013-9241-z

**Published:** 2014-01-07

**Authors:** Pasan Fernando, Xuxu Yan, Julia Lockwood, Yin Duan, Lihui Wei, R. Glenn Wells, Corinne Bensimon, Wayne M. Mullett, Terrence Ruddy

**Affiliations:** 1 Nordion, 447 March Road, Ottawa, ON K2K 1X8 Canada; 2 Division of Cardiology, Department of Medicine, Faculty of Medicine, University of Ottawa Heart Institute, 40 Ruskin Street, Ottawa, ON K1Y 4W7 Canada; 3 Canadian Molecular Imaging Centre of Excellence, University of Ottawa Heart Institute, 40 Ruskin Street, Ottawa, ON K1Y 4W7 Canada; 4 Department of Cellular and Molecular Medicine, Faculty of Medicine, University of Ottawa, Ottawa, ON K1H 8M5 Canada

**Keywords:** Rotenone, Pesticide, Subacute toxicity, Myocardium, Perfusion, Radiotracer, SPECT molecular imaging

## Abstract

Myocardial perfusion scintigraphy is a valuable clinical tool for assessing coronary blood flow deficits in patients. We recently synthesized a new iodinated compound (^123^I-CMICE-013) based on rotenone and showed that it has excellent performance as a radiotracer for myocardial perfusion imaging. Here, we describe the cellular toxicity and subacute toxicity of CMICE-013 in rats. Cultured hepatocytes displayed sensitivity to rotenone but not CMICE-013 at equimolar concentrations. Following i.v. injection of CMICE-013 for 14 days, body weight, ambulation, behavior, grooming, guarding (abdominal, muscular), pale conjunctivae, and food intake were observed. Biochemical, hematological, and histopathological changes in tissues (heart, liver, kidney, spleen, lung, and brain) and echocardiography at pre- and post-dosing were also examined. All animals responded well to the daily injections of CMICE-013 and showed no mortality or adverse reactions with respect to the parameters above. Subacute i.v. injections at high- (5 μg/kg) and low (1 μg/kg)-dose levels did not result in any significant changes to either biochemical or hematological parameters and no detectable changes in histopathology compared to the vehicle or untreated animals. Echocardiographic analyses, including the measurements of cardiac function and anatomy (wall thickness, left atrial size, and left ventricular mass), were not different at pre- versus post-dose measures and were not different compared to the vehicle or untreated animals. Our observations in small animals reveal that CMICE-013 induces minimal toxicity when delivered intravenously for 14 days.

## Introduction

Coronary artery disease is the major cause of early morbidity and mortality in North America and worldwide. The early detection of heart disease is of paramount importance in risk stratification for heart patients. As such, myocardial perfusion scintigraphy has become a favored tool for the noninvasive detection of obstructive coronary artery disease in patients with presumptive perfusion deficit. Thallium-201 was the first radionuclide used universally for myocardial perfusion scintigraphy [23, 26]. The medical community has since adopted numerous agents for the noninvasive assessment of myocardial perfusion. Currently, the more frequently used agents are the Technetium-99 m compounds sestamibi and tetrofosmin. Differential uptake of these tracers in cardiomyocytes as determined by blood flow permits the discrimination between normal and ischemic myocardium.

Accumulation of ^201^Thallium-chloride, ^99m^Tc-sestamibi, and ^99m^Tc-tetrofosmin in the cell is related to membrane potential [4, 9, 19, 25]. Localization of these tracers appears to be determined by regional intracellular ionic charge. The negative mitochondrial membrane potential facilitates the sequestration of sestamibi and tetrofosmin to the mitochondria, whereas thallium, a K^+^ analog, resides predominantly in the cytosolic compartment. We recently designed a new radiotracer CMICE-013, which is a rotenone analog with modifications at the 6′ and 7′ carbons. With CMICE-013, rotenone is iodinated at the 7′ carbon. A hydroxyl group is added to the 6′ carbon, and the 7′ double bond is reduced to a single bond. This renders CMICE-013 more hydrophilic than rotenone. CMICE-013 is suitable for SPECT myocardial perfusion scintigraphy and was shown to have improved characteristics relative to the popular Tc-99m radiotracers [33].

Rotenone [(2R,6aS,12aS)-1,2,6,6a,12,12a-hexahydro-2-isopropenyl-9,9-dimethoxychromeno [3,4-b] furo(2,3-h)chromen-6-one] is a naturally occurring toxin that is structurally related to the isoflavone family of compounds. Rotenone is derived from the roots of select tropical plants of the Leguminosae family, namely cube or barbasco (*Lonchocarpus utilis* and *L. urucu*), derris (*Derris elliptica*), rosewood (*Tephrosia* spp.), and Rabbit’s pea (*Dalbergia paniculata*). Root extracts from these plants have been used for centuries as a pesticide in South America and Southeast Asia [17].

Rotenone, as with many other pesticides, functions at the level of mitochondrial respiration by blocking the oxidation of reduced nicotinamide adenine dinucleotide (NADH) to NAD. Recent evidence suggests that these compounds bind within a cavity situated at the interface of 49-kDa and PSST subunits of mitochondrial complex I [29, 32]. Interestingly, the region of binding is specific despite the structural diversity among complex I-inhibiting pesticides [29]. The loss of NAD results in a failure to oxidize key substrates of the tricarboxylic acid cycle. Additional effects of rotenone include alterations to fatty acid metabolism and aerobic oxidation [8, 13, 15, 35], and excessive doses of rotenone can cause neurotoxicity resulting in Parkinson’s-like phenotypes [7, 27, 28].

Given the high affinity of rotenone for mitochondrial complex I and that cardiomyocytes are highly enriched with mitochondria, we have designed a series of rotenone derivates for use in myocardial perfusion scintigraphy. In particular, a candidate derivative CMICE-013 was recently employed in a series of animal studies. This compound had high specificity for the myocardium, thus signifying its potential as a perfusion tracer for assessing coronary artery disease (CAD) [33]. In view of the potent pesticidal mechanism of rotenone, we report here a comprehensive toxicological evaluation of the rotenone derivative CMICE-013 in rats within the context of a myocardial perfusion tracer.

## Methods

### CMICE-013 Preparation

In previous studies where CMICE-013 was evaluated as a myocardial perfusion tracer, radiolabeled CMICE-013, i.e., ^123^I-CMICE-013, was employed. In the present study, we used the non-radioactive analog ^127^I-CMICE-013. The preparation and characterization of ^127^I-CMICE-013 (herein referred to as CMICE-013) was conducted under sterile and aseptic conditions as described [33]. Briefly, rotenone in trifluoroacetic acid (TFA) was mixed with NaI in 0.1 M NaOH. A 15 mg/mL solution of iodogen (1,3,4,6-tetrachloro-3α,6α-diphenylglucoluril) in TFA was added drop wise with stirring. The reaction mixture was heated to 60 °C with constant mixing for 45 min and then concentrated with a rotary evaporator (Buch, R-210). Milli-Q water and dichloromethane were added sequentially to the mixture to facilitate a liquid–liquid extraction. The organic phase was collected and dried over sodium sulfate. Following evaporation, the crude product was purified on a preparative reverse-phase column (Phenomenex Luna C18(2)) and eluted with Milli-Q water and 95 % ethanol. The purified product was collected and evaporated to complete dryness.

The maximum formulated dose of CMICE-013 suitable for clinical use was 5.0 μg/kg in 5 % ethanol/10 mM sodium acetate (pH 5). As such, a low and high dose, 1.0 and 5.0 μg/kg, respectively, was prepared for animal studies and their concentrations were determined by HPLC. The final low- and high-dose CMICE-013 products were dispensed into crimped vials and stored for a single thaw use at −80 °C.

### Serum stability measurements

Approximately 0.9 μg/L of CMICE-013 or rotenone in 5 % ethanol was added to an equal volume of rat serum or PBS and incubated at 37 °C for various times with mixing at 400 rpm. An equal volume of 95 % ethanol (1:1:1 compound:serum/PBS:ethanol) was added and the reaction was centrifuged at 4,000 rpm for 5 min at room temperature. An aliquot of each sample was filtered through a 10 K MWCO centrifugal filter (Pall, Port Washington, NY) at 14,000 × *g* for 25–30 min at 12 °C. The filtrate was analyzed by reverse-phase HPLC (Phenomenex Luna C18(2), and the UV trace at 290 nm was integrated to examine the signal peak for either CMICE-013 or rotenone. The percentage peak area was calculated using Empower 2 software (waters) and plotted for each time point.

### In Vitro Hepatotoxicity Assays

Normal human hepatocytes (THLE-3, ATCC, Manassas, VA) were maintained in BEGM media with supplements from the BEGM Bullet Kit (CC3170, Lonza/Clonetics, Walkersville, MD) excluding gentamycin/amphotericin and epinephrine and including additional 5 ng/mL of epidermal growth factor (Life Technologies, Burlington, ON), phosphoethanolamine (70 ng/mL, Sigma-Aldrich, St. Louis, MO), and fetal bovine serum (10 %, Life Technologies). Cells were plated onto 96-well plates precoated with 0.01 mg/mL fibronectin, 0.03 mg/mL bovine collagen type I, and 0.01 mg/mL BSA (Sigma-Aldrich). Cells were incubated for 24 h with either rotenone, CMICE-013, or vehicle (5 % ethanol/10 mM sodium acetate), and cell viability was determined using a colorimetric Cell Cytotoxicity Assay Kit (Abcam, Cambridge, MA) by monitoring the change in absorbance at 570 and 605 nm on a BioTek Synergy HT plate reader.

LDH activity was determined using an LDH Cytotoxicity Assay Kit (Pierce, Rockford, IL). Approximately 2 × 10^4^ THLE-3 cells were seeded onto each well of a 96-well clear tissue culture plate precoated as above. Cells were allowed to adhere overnight and then treated with varying concentrations of either rotenone, CMICE-013, or vehicle for 6 h. The assay was then conducted as per the manufacturer’s instructions, and the absorbance at 490 and 680 nm was determined (BioTek Synergy HT).

Mitochondrial membrane potential was determined using the TMRE Mitochondrial Membrane Potential Assay Kit (Abcam). THLE-3 cells at approximately 2 × 10^4^ cells/well were seeded onto a black walled, clear bottom 96-well culture plate precoated as above. Cells were allowed to adhere overnight and then treated with varying concentrations of either rotenone, CMICE-013, or vehicle for 6 h. TMRE-based mitochondrial accumulation was measured on a BioTek Synergy HT plate reader in fluorescence mode.

### Animals

Male and female Sprague–Dawley rats (Charles River, St-Constant, PQ) at pre-dose weights of approximately 250–300 g (female) and 375–430 g (male) were acclimated for at least 1 week prior to experimentation. Animals were housed in groups of two at 20–24 °C and 30–50 % rh under a 12-h diurnal light cycle and were provided food and water ad libitum (Harlan Teklad Certified Global Rodent Diet, Montreal, PQ). All animal procedures were approved by the University of Ottawa Animal Care Committee and in accordance with the guidelines and regulations stated by the Canadian Council on Animal care.

### Animal Assignments and Dosing

Using an operator-blinded design, animals were randomly assigned to saline, vehicle (5 % ethanol/10 mM sodium acetate), 1 μg/kg CMICE-013 or 5.0 μg/kg CMICE-013 (*N* = 8 for all groups). A single daily dose was administered intravenously via the tail vein using alternate left and right sides of the tail on each day of injection in order to minimize scaring and irritation at the injection site.

Cage-side observations including weight, ambulation, behavior (bright, alert, responsive), signs of lethargy, ruffled fur, lack of grooming, guarding (abdominal, muscular), pale conjunctivae, and decreased food intake were monitored daily throughout the study.

### Echocardiography

Echocardiography was assessed prior to dosing (baseline) and again after day 14 for each animal. Animals were sedated under 2–3 % isofluorane for a maximum of 30–45 min during the echocardiographic acquisition. Measurements were taken using Vevo 770 software (VisualSonics Inc., Amsterdam) and included parasternal long-axis assessment (B-mode) and short-axis measures (M-mode) of left ventricular diastolic and systolic volumes, stroke volume, cardiac output, wall thickness, ejection fraction, and fractional shortening. Left atrial size was measured in M-mode and averaged over five views.

### Necropsy and Histopathology

Following the final ultrasound scan, animals were euthanized by CO_2_ asphyxiation and a necroscopic examination was performed on each animal, which included a gross examination and macroscopic organ assessment with the preservation of blood, heart, liver, lung, kidney, spleen, and brain. Tissue samples were immediately preserved in 10 % buffered formalin. Blood smears, EDTA blood, and serum were processed and analyzed for biochemistry and complete blood count (Animal Health Laboratory, University of Guelph, Guelph ON). Following a minimum of 48-h fixation, the tissues were embedded in paraffin and sections were cut at 7.0 μm and stained with hematoxylin and eosin (H&E) to examine cellular morphology and intracellular structures.

### Statistical Evaluations

Analyses were completed using a two-way analysis of variance with a Bonferroni post hoc comparison of means. The complete blood count and blood biochemistry results are shown per gender and compared in a similar manner. Echocardiography results were analyzed using a paired two-way analysis of variance comparing gender at pre- and post-treatment across treatment groups.

## Results

### Compound Stability

Rotenone was stable in PBS but showed significant breakdown in rat serum at 37 °C. After 30 min in serum, approximately 75 % of rotenone remained intact relative to rotenone in PBS (*p* < 0.001). This reduced to approximately 35 % after 240 min (*p* < 0.0001). In contrast, CMICE-013 had weak stability in PBS and extremely poor stability in rat serum (*p* < 0.0001). By as early as 15 min, almost half of the CMICE-013 was degraded relative to pre-incubation. After 240 min in serum at 37 °C, less than 10 % of CMICE-013 was intact. Despite the weak stability of both compounds, rotenone was more stable after 240 min relative to CMICE-013 (*p* < 0.0001).

### In Vitro Cytotoxicity

**Fig. 1:**
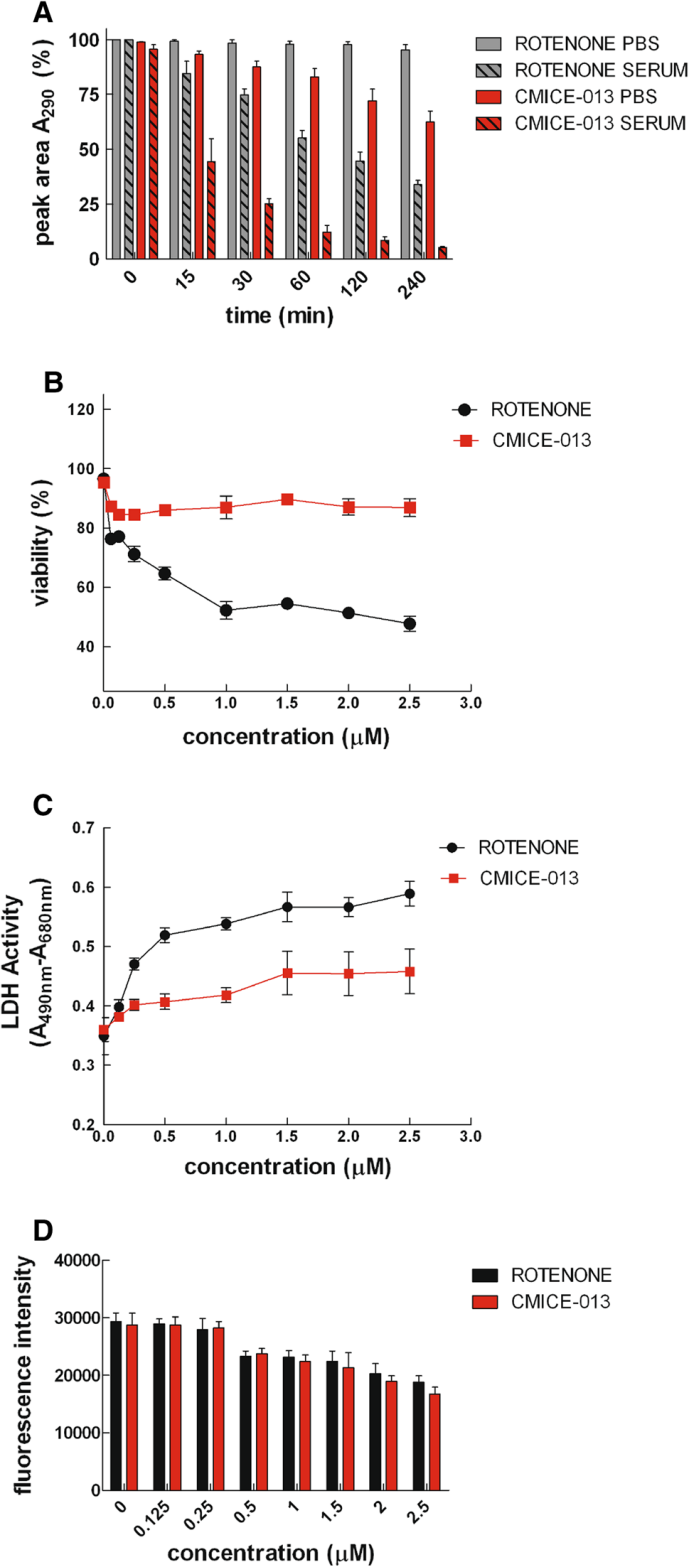
Stability and cellular toxicity of rotenone and CMICE-013. **a** Rotenone and CMICE-013 were incubated in PBS or rat serum and assessed by reverse-phase HPLC. Shown are the percent peak areas based on the HPLC chromatogram for each sample at the respective time point. Values are expressed as the mean ± SEM from 3 independent repetitions per time point for each sample. **b** THLE-3 cells were incubated with either rotenone or CMICE-013 at varying concentrations for 24 h. Viability was expressed as a percentage of the total cells in the vehicle-treated condition. Values are mean ± SEM from 5 independent assays for each compound. **c** Lactate dehydrogenase activity in serum following a 6-h incubation of THLE-3 cells with rotenone or CMICE-013. Values are mean ± SEM from 4 independent assays for each compound. **d** Mitochondrial membrane potential as indicated by the sequestration of TMRE by active mitochondria after 6 h of incubation with either rotenone or CMICE-013 was measured by fluorescence spectroscopy. Values are mean ± SEM from 3 independent assays for each compound

THLE-3 cells demonstrated sensitivity to increasing concentrations of rotenone, whereas minimal cytotoxicity was observed at similar concentrations of CMICE-013. Approximately 85–90 % of cells remained viable with up to a maximal concentration of 2.5 μM CMICE-013. whereas a 50 % viability was found after treatment with 1.0 μM rotenone (*p* < 0.0001) (Fig. 1).

Serum LDH activity increased with elevated concentrations of either rotenone or CMICE-013 (*p* < 0.001). At 1.5 μM CMICE-013, no further changes in LDH activity were found. Overall, rotenone induced higher LDH activity at concentrations above 0.5 μM (*p* < 0.01).

The accumulation of tetramethylrhodamine ethyl ester (TMRE) in the mitochondria was used to assess mitochondrial membrane potential. Although there was a significant effect of concentration on mitochondrial membrane potential (*p* < 0.001), the effect of either rotenone or CMICE-013 was not different.

### Cage-Side Observations and Animal Weight

Rats administered with 1 or 5 μg/kg CMICE-013 failed to show signs of abnormal behavior or discomfort during and several hours after administration. Animals displayed normal ambulation and remained alert and responsive immediately after injection. Throughout the 14-day period, animals did not show signs of guarding and appeared to maintain normal grooming and eating behaviors. Mortality was not observed during the 14-day treatment with CMICE-013 in either sex.

**Fig. 2:**
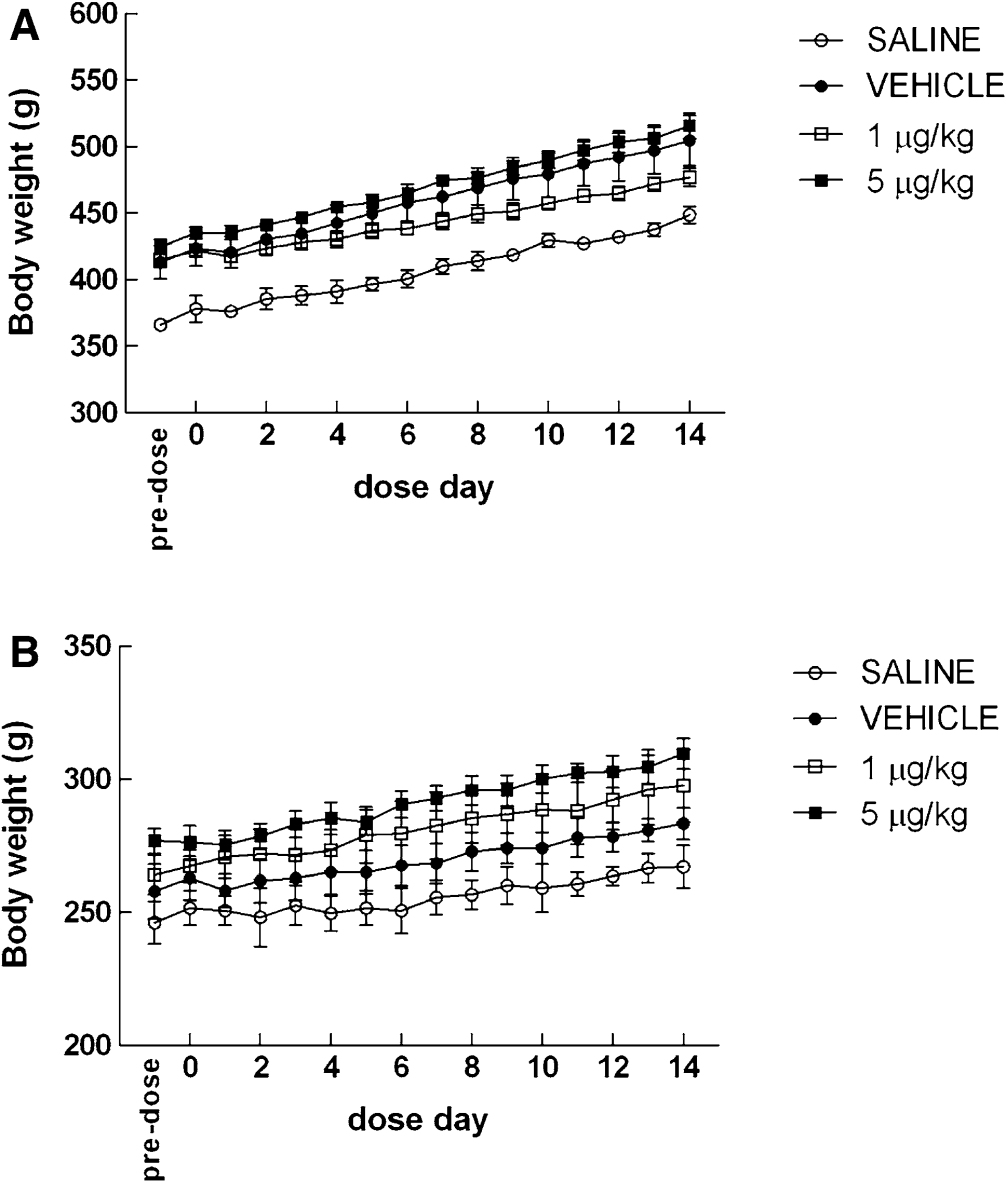
Effect of CMICE-013 on animal body weight. Body weight was measured daily for both male (**a**) and female (**b**) rats. Values are mean ± SEM of 8 rats in each group

Both male and female rats across all treatment groups continually gained body weight throughout the duration of the study (Fig. 2). Despite the positive total weight gain over 14 days, animals that received saline had significantly lower body weights relative to the vehicle and treatment groups (*p* < 0.001 for both males and females).

### Hematological Analyses

Hematological indices such as red and white blood cell number, hematocrit, mean corpuscular volume, mean platelet volume, total serum protein, lymphocyte, and monocyte counts were not significantly different from control and vehicle with CMICE-013 treated rats after 14 days (Table 1).

**Table 1:** Effect on hematological parameters after 14-day i.v. administration of CMICE-013

	Control		Vehicle		1 μg/kg		5 μg/kg	
	Male	Female	Male	Female	Male	Female	Male	Female
RBC (× 10^12^/L)	8.95 ± 0.35	7.95 ± 0.45	7.77 ± 0.07	8.02 ± 0.24	8.50 ± 0.30	7.78 ± 0.23	8.10 ± 0.16	7.90 ± 0.21
Hb (g/L)	164 ± 2.33	160 ± 2.65	152 ± 1.73	152 ± 3.24	160 ± 2.84	146 ± 3.33	159 ± 2.38	148 ± 5.17
RCDW (%)	11.2 ± 0.3	11.5 ± 0.1	12.1 ± 0.3	11.2 ± 0.2	12.4 ± 0.2	11.7 ± 0.2	11.8 ± 0.6	11.8 ± 0.7
WBC (× 10^9^/L)	11.60 ± 1.55	10.70 ± 1.20	8.35 ± 0.31	8.54 ± 0.78	12.80 ± 1.15	13.90 ± 1.24	9.58 ± 0.45	8.93 ± 0.92
Hct (L/L)	0.540 ± 0.020	0.480 ± 0.030	0.500 ± 0.005	0.490 ± 0.009	0.528 ± 0.007	0.475 ± 0.013	0.518 ± 0.007	0.483 ± 0.019
MCV (fL)	65.5 ± 0.5	60.5 ± 0.5	64.0 ± 1.2	61.4 ± 0.8	62.0 ± 1.6	61.3 ± 0.5	64.0 ± 1.1	60.7 ± 0.7
MCH (pg)	20 ± 0.1	19.0 ± 0.1	19.7 ± 0.3	19.0 ± 0.3	18.8 ± 0.5	18.8 ± 0.3	19.8 ± 0.5	18.7 ± 0.3
MCHC (g/L)	303 ± 4	316 ± 2	304 ± 1	310 ± 3	304 ± 3	307 ± 3	308 ± 3	306 ± 4
Platelets (10^9^/L)	700 ± 168	741 ± 61	758 ± 55	739 ± 100	720 ± 70	829 ± 174	797 ± 50	784 ± 105
MPV (fL)	13.0 ± 1.3	11.2 ± 0.8	11.6 ± 0.6	11.3 ± 0.7	11.4 ± 0.6	10.9 ± 0.5	10.8 ± 0.5	11.4 ± 0.2
T.S. Protein (g/L)	76 ± 4	77 ± 1	77 ± 1	78 ± 1	76.8 ± 1	76 ± 2	74.4 ± 1	77 ± 2
SGN (× 10^9^/L)	0.460 ± 0.060	0.510 ± 0.100	0.513 ± 0.116	0.608 ± 0.076	0.588 ± 0.076	0.658 ± 0.057	0.526 ± 0.063	0.690 ± 0.046
Lymph (× 10^9^/L)	10.90 ± 1.69	9.07 ± 0.93	10.60 ± 0.44	8.46 ± 0.87	12.30 ± 0.72	10.6 ± 1.72	8.48 ± 0.42	11.7 ± 2.18
Mono (× 10^9^/L)	0.290 ± 0.010	0.295 ± 0.015	0.257 ± 0.023	0.272 ± 0.028	0.295 ± 0.032	0.288 ± 0.028	0.306 ± 0.030	0.253 ± 0.029

Values are expressed as mean ± SEM of 8 rats in each group

*RBC* red blood cell, *Hb* hemoglobin, *RCDW* red cell distribution width, *Hct* hematocrit, *MCV* mean corpuscular volume, *MCH* mean corpuscular hemoglobin, *MCHC* mean corpuscular hemoglobin concentration, *MPV* mean platelet volume, *T.S Protein* total serum protein, *SGN* segmented neutrophil count, *Lymp* lymphocyte, *Mono* monocyte

### Biochemical Analyses

After 14 days of treatment, the standard biochemical parameters including total protein, albumin, globulin, urea, glucose, cholesterol, and total bilirubin were not different among the treatment groups. A slight increase in blood glucose concentration appeared with elevated CMICE-013 concentrations for both male and female rats but was not significant (*p* < 0.05) (Table 2). Key markers of renal and hepatic function including alkaline phosphatase, alanine aminotransferase, aspartate aminotransferase, and gamma-glutamyl transaminase remained unchanged after 14 days of CMICE-013 treatment (Table 2).

**Table 2:** Effect on biochemical parameters after 14-day i.v. administration of CMICE-013

	Control		Vehicle		1 μg/kg		5 μg/kg	
	Male	Female	Male	Female	Male	Female	Male	Female
Total protein (g/L)	69.5 ± 2.5	70 ± 3.0	67 ± 1.7	71.2 ± 1.2	68 ± 2.0	69 ± 1.5	66.6 ± 0.6	71 ± 0.6
Albumin (g/L)	40.5 ± 1.5	49.5 ± 3.5	41.0 ± 0.6	47.0 ± 1.3	41.8 ± 1.4	45.0 ± 1.4	43.6 ± 1.1	45.3 ± 1.2
Globulin (g/L)	22.50 ± 0.50	20.50 ± 0.50	27.02 ± 1.53	24.20 ± 0.80	26.75 ± 1.44	24.13 ± 1.47	24.21 ± 0.49	23.67 ± 0.33
Urea (mmol/L)	5.05 ± 0.25	5.25 ± 0.15	6.27 ± 0.09	6.42 ± 0.35	5.83 ± 0.48	6.03 ± 0.37	5.20 ± 0.22	6.33 ± 0.32
Glucose (mmol/L)	8.05 ± 0.91	11.25 ± 0.55	8.73 ± 0.42	11.96 ± 0.56	9.48 ± 0.34	14.48 ± 0.87	10.82 ± 0.73	15.03 ± 1.24
Cholesterol (mmol/L)	2.725 ± 0.195	2.745 ± 0.085	2.921 ± 0.163	2.878 ± 0.157	2.830 ± 0.270	2.445 ± 0.239	2.454 ± 0.092	2.447 ± 0.232
Total bilirubin (μmol/L)	2.50 ± 0.50	3.01 ± 0.32	2.67 ± 0.33	2.50 ± 0.50	3.75 ± 0.48	2.75 ± 0.25	3.20 ± 0.20	2.67 ± 0.33
Alkaline phos (U/L)	165.0 ± 8.3	131.5 ± 5.6	183.3 ± 5.7	118.2 ± 4.0	186.8 ± 11.6	126.5 ± 6.1	175.0 ± 6.7	124.7 ± 5.2
ALT (μmol/L)	74.01 ± 6.02	73.04 ± 2.41	74.33 ± 4.84	68.60 ± 6.32	71.25 ± 2.72	63.25 ± 0.85	65.60 ± 4.39	70.67 ± 8.67
AST (U/L)	172.5 ± 6.5	161.5 ± 6.8	165.7 ± 6.3	161.0 ± 7.3	176.0 ± 7.9	163.5 ± 8.6	166.4 ± 5.6	158.7 ± 6.5
GGT (mmol/L)	0	0	0.33 ± 0.13	0	0.25 ± 0.13	0.50 ± 0.11	0	0

Values are expressed as mean ± SEM of 8 rats in each group

*Alkaline phos* alkaline phosphatase, *ALT* alanine transferase, *AST* aspartate transferase, *GGT* gamma-glutamyl transaminase

### Organ Weights and Histology

**Fig. 3:**
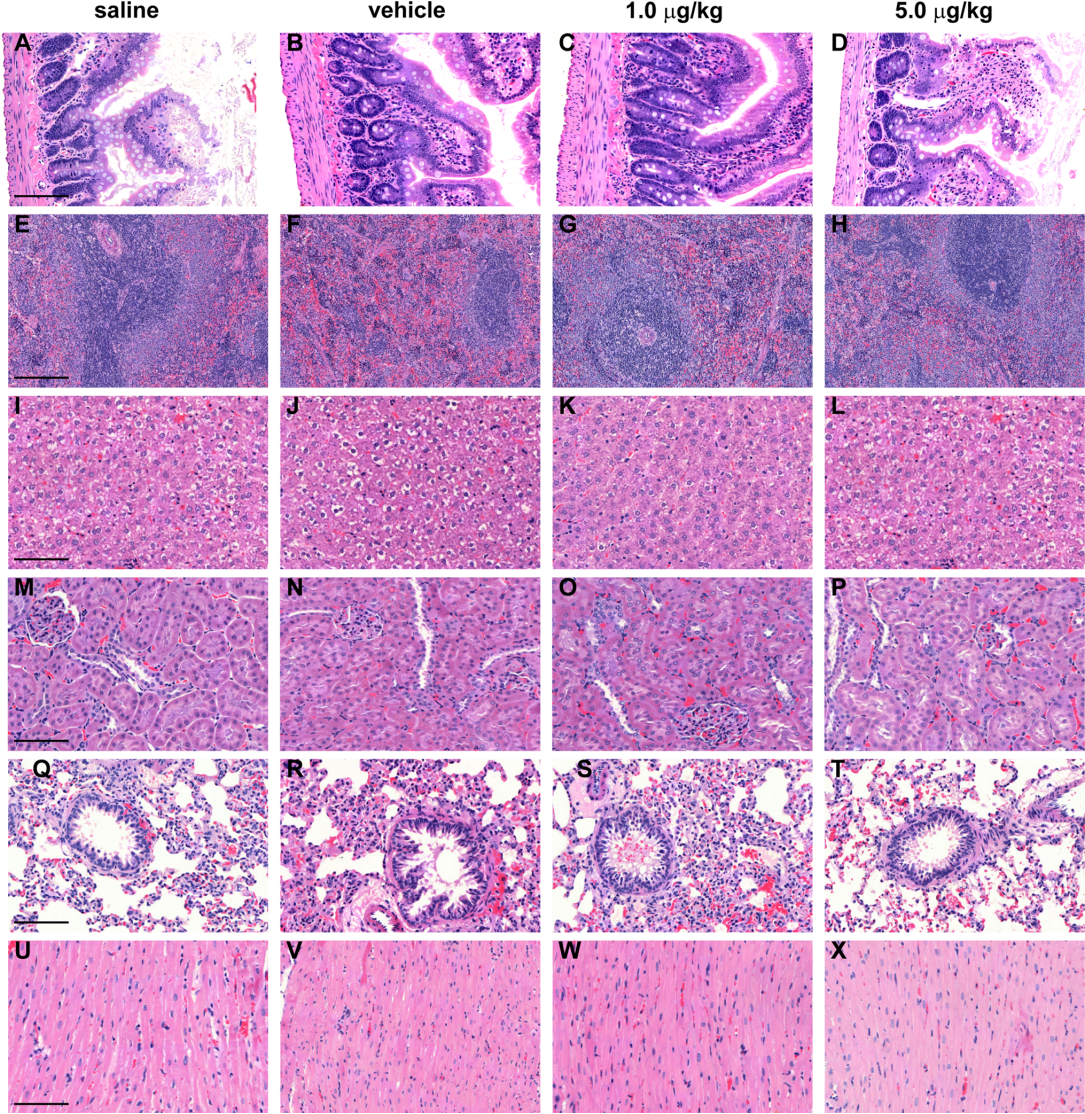
Histopathological analysis of select tissues after 14 days of subacute i.v. administration of CMICE-013. Histopathological micrographs of large intestine (**a**–**d**), spleen (**e**–**h**), liver (**i**–**l**), kidney (**m**–**p**), lung (**q**–**t**), and heart (**u**–**x**). Figures are representative of micrographs from tissue of three animals for each condition. *Scale bar* 100 μm

Gross examination of isolated heart, liver, kidney, and spleen did not reveal any abnormalities or differences in their mean weights. Organ weights expressed relative to animal body weight were compared in order to normalize for variances in size due to gender (Table 3). Overall, histological examination using H&E staining did not reveal differences in organs between treatment groups. The intestinal layers of the large intestine appear intact and unchanged between treatment groups (Fig. 3). Examination of the spleen across treatments shows that the connective tissue appears dense with normal lymphatic nodules. Hepatocytes have normal architecture and show granulated cytoplasms. In addition, the kidneys display regular tubules and glomeruli with normal architecture across all treatment groups. The intramural coronary arteries within the lung displayed some evidence of edema in all treatment groups but this was not a predominant finding overall. The myocardium of the left ventricle displayed regular striated myocytes with nuclei that were normal in appearance.

**Table 3:** Isolated organ weights after 14-day i.v. administration of CMICE-013

	Control		Vehicle		1 μg/kg		5 μg/kg	
	Male	Female	Male	Female	Male	Female	Male	Female
Heart/bw	0.375 ± 0.015	0.475 ± 0.065	0.390 ± 0.015	0.484 ± 0.034	0.425 ± 0.026	0.498 ± 0.042	0.432 ± 0.022	0.4667 ± 0.027
Liver/bw	4.140 ± 0.090	4.300 ± 0.114	3.973 ± 0.125	3.152 ± 0.734	3.795 ± 0.065	3.152 ± 0.692	3.98 ± 0.191	3.901 ± 0.147
Kidney/bw	0.755 ± 0.055	0.680 ± 0.036	0.713 ± 0.023	0.650 ± 0.111	0.675 ± 0.025	0.754 ± 0.013	0.795 ± 0.024	0.717 ± 0.026
Spleen/bw	0.170 ± 0.010	0.213 ± 0.003	0.218 ± 0.013	0.238 ± 0.015	0.210 ± 0.010	0.234 ± 0.011	0.260 ± 0.025	0.247 ± 0.020

Organ weights are expressed relative to body weights (bw) for each animal. Values are mean ± SEM of 8 rats in each group

Neuronal degeneration or cytoplasmic inclusions in coronal sections of the substantia nigra pars compacta after a 14-day treatment with CMICE-013 in rats was not evident. Moreover, immunohistochemical analyses of coronal cross sections were negative for the Parkinson’s disease markers α-synuclein and tau (data not shown). Indeed, the absence of a Parkinson’s-like phenotype is a favorable finding for the purposes of a diagnostic tracer to assess myocardial perfusion.

### Echocardiography

**Fig. 4:**
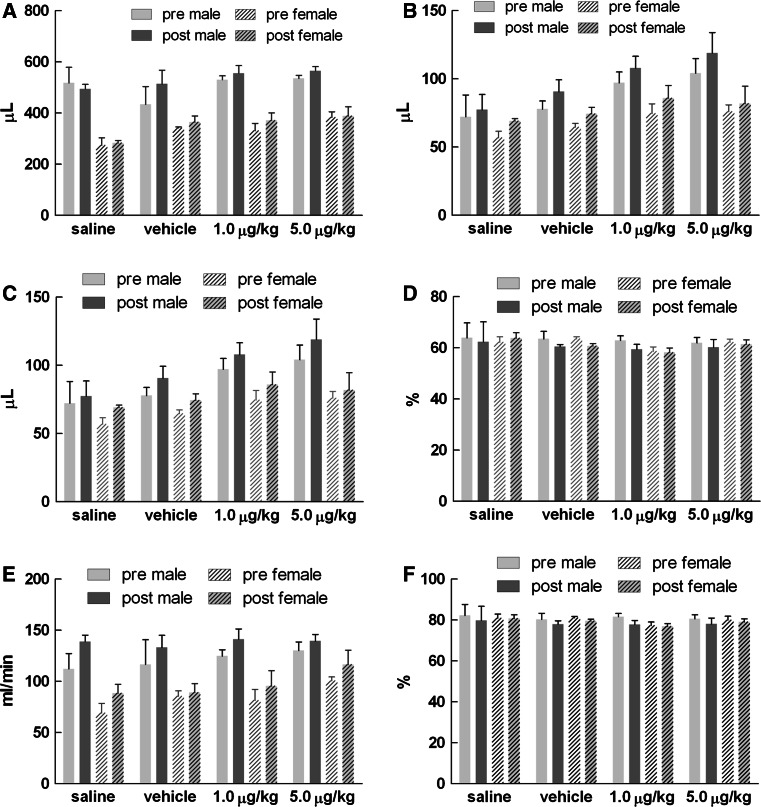
Echocardiography-based functional assessment at pre-dose and after 14 days of subacute dosing of CMICE-013. **a** Diastolic volume, **b** systolic volume, **c** stroke volume, **d** fractional shortening, **e** cardiac output, **f** ejection fraction. Values are mean ± SEM of 8 rats in each group

**Fig. 5:**
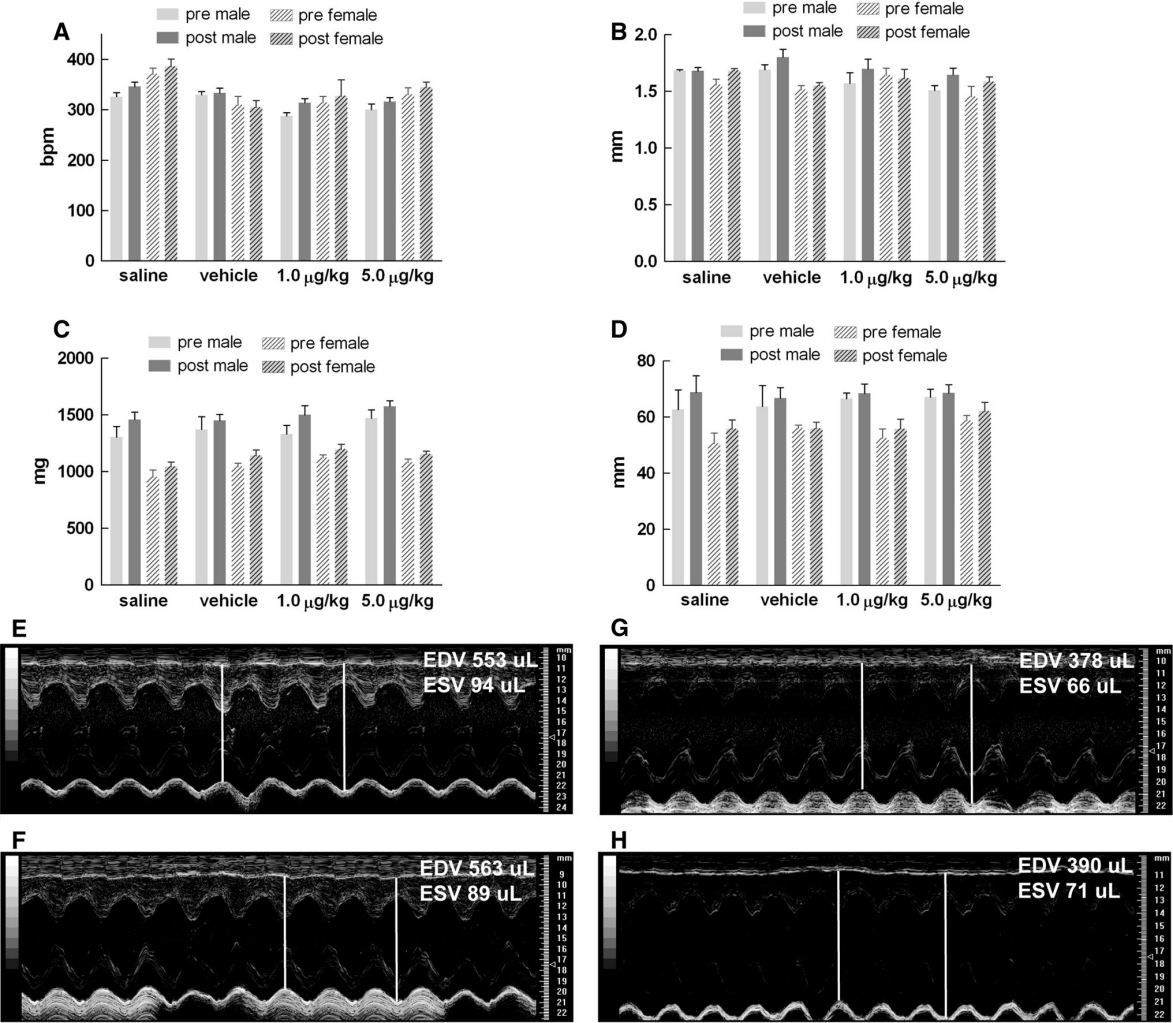
Echocardiography-based anatomical assessment at pre-dose and after 14 days of subacute dosing of CMICE-013. **a** Heart rate, **b** wall thickness, **c** left ventricle mass, **d** left atrial size. Values are mean ± SEM of 8 rats in each group. Representative M-mode sonograms of the left ventricle from a male rat at pre- (**e**) and 14-day post-dose (**f**) and female at pre- (**g**) and 14-day post-dose (**h**) with 5.0 μg/kg/day of CMICE-013. The calculated left ventricular end-diastolic volume (EDV) and left ventricular end-systolic volume (ESV) based on the wall motion depth tracing is shown for each image

Echocardiography compared at pre-dosing and after 14 days of treatment did not reveal any significant changes in cardiac function or anatomy. As expected, gender differences were noted in some variables where males showed elevated systolic and diastolic volumes, stroke volume, and cardiac output (Fig. 4). In addition, males also had higher LV mass and left atrial size relative to females (Fig. 5). The observed differences in male rats are likely due to their larger body mass; however, differences between pre- and post-dose measures within gender were not evident.

## Discussion

Myocardial radio-perfusion tracers are able to provide valuable clinical information for diagnosing perfusion deficits. The predominant mechanism of action for the currently available perfusion radiotracers is through cytoplasmic or mitochondrial accumulation following membrane transport. Although this mode of cellular uptake is sufficient, it is often not as effective as molecules that have direct binding or high-affinity interaction with cellular components. ^99m^Tc-based perfusion compounds are known to associate with the mitochondria; however, it is not yet clear whether they bind specifically or are merely held within the mitochondria. Given the high concentration of mitochondria within the myocardium, the binding of rotenone to mitochondrial complex I allows a more potent and specific mechanism for myocardial perfusion tracers. In a previous study, we synthesized a derivative of rotenone, 6′-hydroxy-7′-iodo-rotenone (CMICE-013), and showed that it had excellent myocardial perfusion characteristics and favorable biodistribution in vivo [33]. Here, we demonstrate that CMICE-013 has an apparently benign toxicological profile in rats when delivered intravenously for 14 consecutive days.

Modification of the 6′ and 7′ positions of rotenone significantly limited the stability of CMICE-013. Serum stability in vitro is likely reflective of stability in vivo, although this needs to be confirmed through further analysis of rodent serum following injection of CMICE-013. Nonetheless, the limited degree of CMICE-013 stability may be the primary mechanism for the lack of toxicity that was observed both in cultured cells and in animals. Interestingly, despite the unstable nature of CMICE-013 in vitro, this compound performs exceptionally well as a perfusion tracer and demonstrates sufficient accumulation in the rodent myocardium [33].

In this study, we used Thle-3 cells as a hepatocyte test model for assessing the cytotoxicity of CMICE-013. Thle-3 hepatocytes are a normal human immortalized liver cell line that have similar morphological, genetic, and biochemical features of primary human hepatocytes [24]. Importantly, these cells are able to metabolize compounds in a similar manner to primary human hepatocytes. The lack of CMICE-013 toxicity relative to rotenone in cultured hepatocytes may be explained by the highly unstable nature of CMICE-013 in serum. Further investigation both in culture and in vivo is needed to explore this mechanism.

Typically, subacute toxicity studies are conducted using a range of doses both above and below the suggested human dose. In the present study, rats were administered an intravenous maximal dose of 5 μg/kg daily for 14 days with CMICE-013. CMICE-013 was designed to be a molecular imaging radiotracer with a projected clinical dose of 0.1 μg/kg delivered as a single intravenous injection, which is well below the dose levels used in the present study. CMICE-013 shares similar physical properties with rotenone in that it is soluble in ethanol at 2 g/L @ 20 °C [17]. In the clinical formulation, CMICE-013 is not soluble beyond 7.3 μg/mL. Given such a low anticipated human dose, we chose to explore the toxicity of this tracer when administered at a dose of 1.0 μg/kg and at the maximum formulatable dose of 5.0 μg/kg.

Over a 14-day period, CMICE-013 at either the low- or high-dose level did not impede the weight gain in either male or female rats. Male rats injected with saline had lower weights than males in the other experimental groups. However, despite using a random group assignment protocol, rats that received saline also had lower baseline weights prior to injection. This suggests that the differences in weight are not likely attributable to the test compound or the excipients. In addition, all animals were observed to be alert and responsive throughout the dosing period with normal ambulation and gait and did not display signs of guarding or deficient grooming, further signifying that at the doses administered intravenously, CMICE-013 did not adversely affect the health of the animals over a 14-day period.

Changes in the hematological system of animals are relatively consistent with human toxicity when studies are conducted for 1 month or less [21]. In our 14-day treatment trial, changes in hematological parameters such as hematocrit, hemoglobin concentration, red and white blood cell number, red cell distribution width, and mean platelet volume were not observed between control and CMICE-013-injected animals. This suggests that the compound did not affect existing red cells and did not interfere with erythropoiesis and platelet formation. The lack of changes in serum protein and lymphocyte and monocyte numbers following CMICE-013 dosing further supports this conclusion.

Biochemical parameters remained unchanged in both males and females following CMICE-013 administration for 14 days. The slight but nonsignificant increase in blood glucose with treatment may arise from the 5 % ethanol excipient in the formulation. Importantly, the serum levels of alanine transaminase (ALT), aspartate transaminase (AST), alkaline phosphatase (ALP), and gamma-glutamyl transferase (GGT) were not affected by CMICE-013 dosing. ALT, AST, ALP, and GGT are key markers of hepatotoxicity and liver injury.

Serum ALT is considered the gold standard indicator of hepatotoxic effects but is often assessed in conjunction with other makers [2, 22]. ALT has two isoforms ALT1 and ALT2 that display differential tissue expression, which may explain the need to assess ALT in relation to other biomarkers of toxicity [16, 37]. Within liver cells, both ALT and AST are localized to the cytoplasm and the mitochondria, however, ALT is found within the periportal region while AST is ubiquitously expressed. The liver has the highest expression of ALT1 but lower levels are also found in skeletal muscle and heart. ALT2 is restricted to skeletal muscle and heart [16]. In comparison, AST has lower levels of expression in the liver and is found more abundantly in skeletal muscle, heart, brain, and kidney. Thus, AST can originate from damaged myocytes as well as hepatocytes, and the ratio of AST:ALT can reveal differences in liver damage relative to other organs [3, 22]. In our studies, AST:ALT was unchanged across treatments for both males and females (*p* < 0.05). These results would suggest that in addition to the liver, CMICE-013 did not induce cytotoxicity in either skeletal- or cardiomyocytes, despite its high affinity for mitochondria and mitochondria rich tissues.

Rotenone is commonly used to model Parkinson’s disease in rats [6, 12, 20]. These studies typically require large systemic doses of rotenone (> 2–3 mg/kg/day) for excessive periods (> 3 weeks) [5, 7]. Bilateral lesions in the striatum with neuronal loss were found after 1 week of rotenone delivery in rats. However, this phenotype emerged using intravenous infusion of rotenone at concentrations of 10–18 mg/kg per day [11]. Nigrostriatal dopmainergic loss was found after intravenous and subcutaneous infusion of 2–3 mg/kg per day of rotenone in rats; however, neurodegeneration did not appear until at least 3 weeks [5, 30]. Moderate doses of rotenone resulted in neurodegeneration of the nigrostriatal dopaminergic pathway after 1 week; however, a chronic sustained delivery via osmotic minipumps was required to bring about this effect [10]. In contrast, degenerated dopaminergic neurons were observed in rats given daily intraperitoneal injections of rotenone at 1.5 and 2.5 mg/kg for 2 months [1]. Clearly, studies that use rotenone to model PD in rodents require significant doses over prolonged periods. Although CMICE-013 is structurally different than rotenone and has attenuated cytotoxicity in cultured cells, it has similar mechanistic properties, i.e., affinity for mitochondrial complex I. Nonetheless, the most obvious explanation for the lack of a PD-like phenotype in our studies is likely attributable to the low doses of CMICE-013 relative to other studies that use rotenone.

Although echocardiography is not a widespread approach in toxicological profiling, it has been used on several occasions to investigate drug-induced cardiotoxicity and is gaining recognition as a reliable tool for assessing the pharmacological effect of drugs [14, 36]. The mechanistic action of rotenone and CMICE-013 may facilitate the perturbation of cardiomyocyte metabolic activity and ultimately affect contractility. However, the echocardiographic analyses did not reveal such an effect. Measurements at baseline and after 14 days of CMICE-013 treatment did not show differences in stroke volume, cardiac output, ejection fraction, and percent fractional shortening, strongly suggesting that hemodynamic function remained unchanged. Furthermore, over a 14-day period, CMICE-013 was unable to induce anatomic changes in the myocardium with respect to wall thickness, left ventricular mass, and left atrial size. These results were also confirmed at the time of necropsy through gross morphological examination and with histological analyses of the myocardium. Conceivably, anatomic changes may require a longer period to manifest. Anatomic changes in the heart following myocardial ischemia/infarction typically appear after 3–4 weeks, and therefore, a longer observation period may be necessary to determine the phenotypic effects on the myocardium with chronic CMICE-013 administration.

Our studies suggest that CMICE-013 is a less toxic variant of rotenone. This may be explained by its attenuated stability in serum but may also be attributed to structural modifications, despite having similar avidity as rotenone for MC-1. Although rotenone toxicity is known to exert effects via the inhibition of mitochondrial activity, cytotoxicity is not exclusively limited to the inhibition of MC-1. In neuroblastoma cells, rotenone induced cytotoxicity through both metabolic perturbation and apoptosis [18]. Rotenone also induced cytotoxicity through the activation of apoptotic signaling pathways [31, 34]. In murine microglia, rotenone induced cytotoxicity through NFκB-associated signaling including the release of TNFα and IL-1β, elevated TNFα and IL-1β mRNA, and increased the phosphorylation of IκB, ERK, JNK, and p38 MAPK [38]. Clearly, rotenone can exert toxicity through mechanisms that are indirect of mitochondrial complex-1 inhibition. Given the differences observed with CMICE-013 relative to rotenone, a comprehensive study examining the structure–activity relationship is warranted within the context of cellular toxicity.

In summary, this study describes the toxicological outcome following intravenous administration of the rotenone derivative CMICE-013 at low (1.0 μg/kg) and high (5.0 μg/kg) doses to rats for 14 consecutive days. Serum proteins and hematological parameters remained unchanged. Echocardiography assessments did not reveal changes in hemodynamics or cardiac anatomy. These observations were further confirmed by the examination of organ and tissue histology. Our results demonstrate that CMICE-013 in its current formulation is safe in rodents. However, additional studies in large animals using both acute and subacute dosing regimens are required in order to claim CMICE-013 safe for clinical applications.
